# The Position of the Ormond Street Hospital

**Published:** 1911-05-27

**Authors:** 


### HOSPITALS OF TO-DAY.

### VI.—THE POSITION OF THE ORMOND STREET HOSPITAL.

The Hospital for Sick Children in Great Ormond Street has passed through a critical twelve months; it has conducted a searching investigation into its administrative methods, and it has shared in the largest triple legacy that has been left to individual hospitals within recent memory. These facts are brought to mind by the advent of the annual meeting, which, if it is allowed to have cognisance of these affairs in anything like full measure, should be one of the most important that the hospital has held. It is certain at any rate that the investigation and the legacy will influence its future development profoundly. The investigation has confirmed the confidence of the hospital's subscribers, whose support, it may be stated on authority, has shown no tendency to diminish, and, as the pages of recent issues of The Hospital can testify, has led to its arrangements being overlooked in such a way as to make administrative errors by which the patients would suffer practically impossible. Mr. Silver's legacy of £25,000 at the time of writing has not yet been relegated, but it may be expected that the new year in the hospital's life will disclose, if not at the meeting itself, the policy of its expenditure.

Before dealing with that, however, it may be well to recall Mr. Silver's relation to the hospital, and once more to place on record the motive which led him to make such an enormous grant to this institution. The Hospital for Sick Children has made a specialty of organised appeals: it has relied with great success upon obtaining the co-operation of a prominent figure in State or Church or literature, and in holding every year a festival dinner, under such leadership. A record of course has been kept, and since 1858, when Charles Dickens lent his eloquence to the hospital, the most famous of these appeals has been that organised by Mr. *Punch* in 1900. Humour in life and letters, therefore, has been a chief agent in engaging public support for the children of this hospital. It was as a member of the staff of *Punch* that Mr. Silver first exerted himself for the institution, and his retirement from journalism in 1870, as The Hospital recorded on December 24 last, was only the beginning of quiet but steady institutional work for this and his other favourite charities. His legacy of £25,000 closes this record as far as the hospital in Great Ormond Street is concerned, and it is safe to prophesy that the mortgage debt, under which the hospital has been labouring, and which has now been reduced approximately to £14,000, will have a first claim upon it.

The contraction of this mortgage debt some few years ago was in one sense a turning-point in the history of the hospital. The circumstances out of which it arose were as follows. By the side of the Hospital for Sick Children was a site nearly as large as the site of the hospital, which provided light and air for all the north side of the building. This was the property of the Roman Catholic authorities responsible for the Hospital of St. John and St. Elizabeth, and on it they proposed to build. Their legal right to do so gave them the whip-hand of the Hospital for Sick Children, for did they put it into force the hospital would have become so unsuited to the treatment of sick persons, and especially of sick children, as to become uninhabitable in a modern medical sense. There was no other course open but to buy the owners out, and this was done for £30,000, a sum which it is idle, at this date, to speak of as excessive. This purchase affected the hospital in two ways: it has improved its internal economy by allowing better accommodation for nurses, which was badly needed, and it has laid on the treasurer the responsibility of paying off the loan contracted in order that it might be made. This loan was arranged through the Prudential Assurance Company at three and a half per cent., on condition that it should be paid off in instalments before July, 1930, according to the scheme which the Charity Commissioners approved. More than half the loan has been paid off already, but no new developments are likely to be undertaken till the whole sum has been repaid.

At the present moment, however, the hospital is in the satisfactory position of enjoying the advantages, the smooth working, of previously made improvements, of which the recently established new out-patient department is the chief. The elaboration of this department with its almoner, inspection and isolation rooms, newly started dental work, casualty department, dispensary and out-patients' hall is the development of a system which was begun under difficulties and cramped space. Now the pathological and $x$-ray departments are housed in the original out-patient receiving rooms, and the nurses' dining-room is the original out-patients' hall. How necessary a system is for dealing with the out-patients at this hospital is obvious from the enormously extended area from which its out-patients come. Not Greater London, not even the Home Counties are the limits from which its patients are drawn. It is practically the consulting centre of England for all diseased children. This means that many of its patients are seen only once, and a special system has been inaugurated whereby not the lady almoner merely, but another officer first, examines their right to treatment and their opportunities to pay. A system is also growing by which the books recording the patients' names and treatments are being kept, so that any applying twice may be recognised and their proper classification may be remembered. Twelve years being the limit up to which children are received, it is hoped eventually to have these records for that period. At present the books for three years have been kept, and it is decided to extend this period to six and then to nine years as may be convenient.

It is curious to notice the results of the agreement which the hospital made with the London County Council, by which it was to receive twenty-four school children a week in the ophthalmic and aural departments, subject to the payment of £150 a year for the salaries of the clinical assistants, as well as two shillings a head for each child treated, no rights of inspection or control being allowed. It has been found in practice that, instead of a tendency for this number to increase, it has been rarely equalled, three or four cases a day having been sent in place of the expected half-dozen. The reason of this, no doubt, has been the want of coincidence between the ages of the children when inspected by the Council and the age limit set by the hospital. As stated above, the hospital receives children up to twelve years of age; the final inspection by the Council takes place at fourteen, so that only the primary and intermediate school inspections affect the hospitals.

It will be seen, then, that the payment of the mortgage debt is the main factor in the situation. But it must be added there is an accumulated deficit of over £5,000. Against this must be set Mr. Silver's legacy. Various smaller matters deserve attention, the principal of which is the absence of electric light in certain wards and nurses' quarters. In other words, the hospital has settled down to steady progress on the improved lines made possible by the purchase of the neighbouring site and by the structural alterations that resulted from it.

